# Comparative ethnographies of medical research: materiality, social relations, citizenship and hope in Tanzania and Sierra Leone

**DOI:** 10.1093/inthealth/ihaa071

**Published:** 2020-11-09

**Authors:** Shelley Lees, Luisa Enria

**Affiliations:** Department of Global Health and Development, London School of Hygiene and Tropical Medicine, 15-17 Tavistock Place, London WC1H 9SH, UK; Department of Global Health and Development, London School of Hygiene and Tropical Medicine, 15-17 Tavistock Place, London WC1H 9SH, UK

**Keywords:** Africa, anthropology, clinical trials, comparative, Ebola, HIV

## Abstract

In this paper we bring together ethnographic research carried out during two clinical prevention trials to explore identities, relations and political imaginations that were brought to life by these different technologies. We highlight the ways in which critical anthropological engagement in clinical trials can help us radically reconsider the parameters and standards of medical research. In the paper we analyse the very different circumstances that made these two trials possible, highlighting the different temporalities and politics of HIV and Ebola as epidemics. We then describe four themes revealed by ethnographic research with participants and their communities but mediated by the specific sociopolitical contexts in which the trials were taking place. In both countries we found materiality and notions of exchange to be important to participants’ understanding of the value of medical research and their role within it. These dynamics were governed through social relations and moral economies that also underpinned challenges to Western notions of research ethics. The clinical trials offered a language to express both disaffection and disillusionment with the political status quo (often through rumours and anxieties) while at the same time setting the foundations for alternative visions of citizenship. Attached to these were expressions of ‘uncertainty and hope’ steeped in locally distinctive notions of destiny and expectations of the future.

## Background

Since the 1990s there has been an expansion of clinical trials in resource-poor contexts in response to emergent disease epidemics such as HIV and Ebola.^1^^–^^6^ Setting-up clinical trials in such contexts raises political economic and social justice questions.^3^^,^^7^^–^^9^ In particular, the focus on participant rights, especially through narrow framings of informed consent and researcher and community relationships, does not take into account broader issues of inequities in wealth and in health status and healthcare resources.^3^^,^^7^^,^^10^

Drawing on ethnographic research we conducted during two clinical trials (MDP 301 and EBOVAC-Salone), established a decade apart, across the African continent (Tanzania and Sierra Leone) and during two different epidemics (HIV and Ebola), we highlight the effect of global and local power dynamics and everyday encounters with biomedicine on the imaginaries of trial participants and their communities. Anthropological engagement with clinical trials provides a critical lens with which to examine the moral and ethical consequences that emerge from the conduct of clinical trials as well as to explore the social, political, cultural and economic dimensions that influence clinical trial participation and longer term legacies of clinical trials on communities.

Anthropological reflections show that technologies are not simply a collection of material objects that exist in isolation from social practice, but are integral to social life and social meaning.^11^^,^^12^ Microbicide gels are a technology of health, intimacy, pleasure and reproduction, as well as medicalisation and the control of sex.^11^^,^^13^^,^^14^ They represent biopower, which denotes power over life, on one hand maximising the forces of the body and on the other a focus on regulation and control of the body.^13^^,^^15^^,^^16^ Vaccine development as part of a system of knowledge and strategies in the eighteenth century to proliferate life and avoid death made the possibility of living a norm rather than a remote possibility.^17^ As a biotechnology they enable control over, and positive transformation of human life, but have also been described as a means of control of populations when vaccine programmes are mandatory.^18^ This notion of biopower may be limited in vaccine trials where individuals make decisions to participate and be vaccinated. In addition, analyses of biopower for vaccine trials ignore agency, ‘self-worry and its modes of expression’.^19^ Instead, we argue that biomedical technologies can transform power and open up choices and possibilities.^20^ Building on existing anthropological studies on clinical trials^5^^,^^7^^,^^21^^–^^24^ in this paper, we propose a comparative approach that allows us to explore common themes in the social meaning of medical research while also showing how these are highly specific to the context, the epidemic and the trial in their manifestation. In our conclusions we reflect on the implications of this comparative approach for the integration of anthropological perspectives in the conduct of clinical trials.

## Situating epidemics in the political context of Tanzania and Sierra Leone

### Postcolonial Tanzania and the HIV epidemic

In 1983, the first cases of AIDS were reported in Tanzania. Over the next 10 y cases continued to rise rapidly, reaching a peak in the mid-1990s. By this time the epidemic had spread from urban to rural areas.^25^ The epidemic coincided with an era of massive social change and the adoption of a neoliberal agenda by the government. Under the Arusha Declaration (Ujamaa) the first president Nyerere had promised a decentralised government and a programme of rural development. With demands for reforms of the structural adjustment programme (SAP) required by the International Monetary Fund, the second president Mwinyi abandoned Ujamaa and conducted a major reform of health policy.^26^^,^^27^ This neoliberal shift in Tanzania created a withdrawal of the state and a growing focus on individual choice.^28^ The commitment to SAPs saw a sharp decline in spending on healthcare with an immediate effect on the ability of the state to provide free and quality health and welfare services.^29^ In response to these crises, the international donor community and local non-governmental organisations (NGOs) stepped in to provide some of the health and welfare previously provided by the state, leading to a rapid growth of the NGO sector. By the mid-2000s,1.6 million people were living with HIV, the majority of whom were women.^30^ In Tanzania, women were disadvantaged in relation to men in access to education, employment and land and property ownership,^31^^,^^32^ and this, as well as biological susceptibility, put them at greater risk of HIV, especially those working in social venues (such as bars), where sexual transactions were common.^33^ With the reduction of government healthcare provision, HIV treatment was only available at NGOs or private health clinics when the trial was set up.

### Postconflict Sierra Leone and the Ebola epidemic

A decade later, an outbreak of Ebola in West Africa captured the international imagination. Sierra Leone was among the worst affected countries, with >14 000 cases reported from 2014 to 2016.^34^ Initial assessments of the epidemic focused on community resistance, bushmeat consumption and traditional practices, however, social scientists identified structural roots.^35^^–^^37^ Ebola was a disaster waiting to happen, the culmination of a particular historical trajectory steeped in the dynamics of an extractive international political economy.^38^ From its key position in the Atlantic slave trade, then a swift transition from abolitionist utopia to British colony, Sierra Leone's economy was designed in aid of international capital. Rapid electoral decolonisation and the legacy of indirect rule in the rural hinterland created significant political challenges for independence in 1961. Colonisers left behind a state with little power and legitimacy among the population.^39^^,^^40^ Following independence, the establishment of a one-party state coincided with a fall in global commodity prices and the introduction of extensive SAPs, including the privatisation of health services. These factors led to the exclusion of a large majority of the population from formal employment, education and healthcare. These trends contributed to the eruption of a civil war that lasted from 1991 to 2002. The war further dilapidated public resources and physical infrastructure.

This legacy, combined with the postwar financing of health services through a patchwork of internationally funded ‘vertical’ programmes, made it extremely challenging to respond to the Ebola outbreak.^38^ As the virus spread, healthcare workers found themselves ‘confronting Ebola with bare hands’.^41^ Meanwhile, citizens’ relations to the national and international institutions that were supposed to support them during the epidemic had been eroded by a long history of extraction and neglect.^42^ Over the course of the epidemic, various interventions, including extensive community engagement efforts supported by anthropological research, worked towards building confidence among affected communities.^43^^–^^46^ It was against this backdrop, in 2015, as the epidemic was beginning to wane, that the Ebola vaccine trials arrived in Sierra Leone.

## Methodology: anthropological engagement with clinical trials

### Microbicides trial in Tanzania (2005 to 2009)

In the early 2000s,  in response to the growing epidemic among women in Africa, there was an international effort to develop microbicide gels, substances which could be used by women, secretly if need be, to protect themselves against HIV infection through vaginal sex. The Microbicides Development Programme (MDP) conducted a phase III, randomised, double-blind, placebo-controlled trial in South Africa, Tanzania, Uganda and Zambia to test the efficacy of microbicide gel.^47^ In Tanzania, a feasibility study found that women working in social venues (bars, restaurants; i.e. local places selling food and alcohol) had a higher prevalence of HIV than the general population and were thus recruited to participate in the trial. The income from working in social venues was under the minimum wage of US$65 per month at the time of the study and many women supplemented their income with transactional relationships at these venues.^14^ The trial included a community liaison team and a social science team. The community liaison team conducted regular participatory dialogue meetings with participant representatives quarterly as well as bi-annual meetings with a stakeholder advisory group.^48^^,^^49^ During the trial SL conducted 4 y of longitudinal research with a team of four local research assistants to understand women's participation in the trial and community perceptions of the trial within the specific historical, political and economic context of Mwanza City. Longitudinal data collection involved ethnographic observations at trial clinics, social venues and community engagement meetings, 99 in-depth interviews with trial participants at 3 time points and 14 focus group discussions.

### Ebola vaccine trial in Sierra Leone (2015 to date)

The declaration of a Public Health Emergency of International Concern in August 2014, Kelly argues, achieved an ‘epistemic shift’ in the imagination of the West African Ebola outbreak. Similarly, the release of models that foreshadowed exponential growth in the epidemic underpinned moral arguments for the international community to act, especially in vaccine development. The ‘sense of urgency that the models conveyed transformed Ebola's value as an object of, and a resource for, global investment’ and the ‘moral outrage and collective responsibility unleashed by this charismatic reworking of the outbreak set the stage for the significance of Ebola vaccines in the response’.^5^ Vaccines were expected to make up for the lost time preventing the epidemic as it spread across West Africa. The first Ebola vaccine trials in the context of an emergency generated heated debates in the scientific community, including whether it was ethical to randomise and what alternative trial designs could be identified to fulfil evidentiary requirements while protecting the rights of participants. Not least due to the enormous challenge of setting up a vaccine trial at emergency speed, and given that the epidemic began to slow down in early 2015, testing for efficacy became impossible in most situations and the trials in Sierra Leone focused on testing for immunogenicity.

The EBOVAC-Salone trial^50^ was established in Sierra Leone's Kambia district in the spring of 2015 and tested the Ad26.ZEBOV/MVA-BN-Filo prime-boost Ebola vaccines. At this time the setting up of the trial coincided with the military-led Operation Northern Push, an effort to suppress a flare up of cases in the district, one of the final hotspots in the country.^51^ In October, a community lottery was held in the Paramount Chief's court to decide which households would be invited to volunteer and a few weeks later the first participants were recruited. Drawing on lessons learned from the social science in Tanzania, SL and LE established a social science research programme to explore experiences of the Ebola outbreak and perceptions of the trial, contextualising these with historical, social, economic and political factors.^52^ LE led the social science with a team of five local research assistants. Data collection methods included ethnographic observations in the trial clinics, within the community and community engagement meetings, 22 in-depth interviews with trial participants at 2 time points, 4 life narrative interviews with trial participants, 7 focus group discussions with trial participants and community members, as well as 30 key informant interviews.

Drawing on the experience of the MDP trial offered insights into how to navigate the role of social scientists in medical research, at once supporting the conduct of the trial by translating contextual observations into operationalisable findings and studying the trial itself as a social practice. We adapted research protocols, designs and methodologies such as participatory rumour-tracking activities in focus group discussions from the work that was carried out in Tanzania. For both case studies, data were transcribed and translated to English and analysed using NVIVO software (QSR International 1999).^53^ Themes were developed as they emerged from the analysis. Comparative analysis was conducted through iterative discussions between LE and SL to identify common themes and context-specific differences.

## Themes

### Materiality and exchange

In Tanzania, the trial provided women with the opportunity to access free, high quality sexual and reproductive care and a financial reimbursement of US$4 for each clinic visit. While government sexual and reproductive health services are supposed to be free, women are often required to pay for medication, thus the free services represented a value.^24^ However, for the women who participated, knowledge was as vital as materiality, acting as an important resource to address uncertainty about their health, the quality of healthcare, their relationships with men and, in particular, about the HIV epidemic. When asked why they had participated, most women said ‘Kujua afya yangu’ (To know my health), suggesting that knowing about health is to be certain about health. The word ‘afya’ also translates to the broader concept of well-being, and knowledge of their HIV status was related to well-being or wholeness (uzima). ‘Knowing’ was also a motive to find out if the gel worked. Their trust in the knowledge they acquired was related to the trial's connections to global actors. As others have noted with regard to participating in a clinical trial, with its global linkages ‘in part substitutes for these other, unreachable or ineffective forms of citizenship or belonging’ [p. 89],^54^ in this way the trial created an enclave of knowledgeable citizens in the context of decaying government health services.^28^

In the EBOVAC-Salone trial, notions of material exchange and the social expectations imbued in them, were manifested in different ways in participants’ reflections on their relationships with the clinic and its staff. Participants spoke of the blood-taking, in the midst of mistrust and fears, as a sacrifice. This was accompanied by expectations of exchange, or material recognition of this sacrifice, such as in requests for the trial to provide food after blood-giving. Longer term expectations were expressed in the belief that ‘there must be something else coming’ or that EBOVAC-Salone surely had ‘some package [of benefits] for participants at the end of the project’ despite an understanding of the participant information.^55^^–^^57^ These expectations reflect the broader dynamics of Sierra Leone's political economy, where sacrificing or ‘suffering for’ more powerful others can be a pathway to recognition and ‘rewards’, even if these are not explicitly promised.^58^

### Social relations and moral economy

In the MDP trial, decisions to participate were influenced, but not determined, by relations with men.^59^^,^^60^ Women's experiences of the gel were articulated through their own broader concerns with sexuality, especially respect and equality in sexual matters. While the use of the gel did not challenge surrounding moral discourses of promiscuity and male control, it contributed to ideas of equality and respect, and to some extent agency, by enabling communication about sexual matters with intimate partners and notions that women's control of sexual pleasure and risk were possible.^61^^,^^62^ Although overtly naming a technology as empowering does not ensure that this will be the case, the women drew on ideas of empowerment, which were transferred from global feminist activists, through the practices and meanings implicit in the trial.^63^ Where condoms remained associated with promiscuity, distrust and immoral behavior,^64^ the gel enhanced both intimacy and trust between women and their partners, as well as sexual desire, pleasure and cleanliness.^62^^,^^65^ While these positive aspects were unexpected, the idea that the technology could address women's sexual oppression and gender inequalities resonated with the women. In short, women's relationships with this technology were grounded in existing habits of pragmatism: as Lock noted, ‘If the *apparent* benefits outweigh the costs to themselves, and if technology serves their own ends, then most women will avail themselves of what is offered’ [p. 2].^66^

New technologies bring rumours as they are often imagined in ways that are never considered by those who develop them.^67^ Thus, it was not surprising that rumours about the gel and its purpose emerged. However, women defied local moral protestations to participate in this trial and situated themselves as ‘knowing’ in contrast to neighbours who were on the outside of the trial. They were selective with the information they shared with their partners or neighbours and rather focused on the benefits of the knowledge acquired for themselves personally. Emboldened by their new acquisition of knowledge women defied ideas about themselves as vulnerable to, or responsible for, the HIV epidemic. The way in which the women dealt with rumours in the community was reflected in their ‘sense of belonging’ to the trial and their positive engagement with the clinical trial staff. These new social relations with trial staff created an alliance of actors addressing women's vulnerability to HIV infection.^68^ While science often makes it difficult for lay people to be involved in the development of technologies,^69^ in the trial women became coproducers of knowledge.

Social relations were also built during the EBOVAC-Salone trial and became central to its operations in ways that were not always visible. Social relations between trial staff and Kambia residents were initially fraught; by hiring staff outside the district, the trial signified a significant influx of ‘strangers’ into town.^70^ This generated tensions due to low formal employment in Kambia, as well as the growing number of relatively well-off people who could rent homes and stimulate the economy. This was particularly meaningful as the trial's set-up phase coincided with the winding down of the outbreak response, which had distorted the local economy by bringing ‘Ebola money’. It had also built resentment for those who were not employed by response.^71^ As the response apparatus retracted, EBOVAC-Salone became the primary employer in town.

The moral economies generated through the arrival of the trial were particularly pronounced for the ‘sons of the soil’, the smaller number of staff recruited from Kambia. These staff showed participants that people they trusted were involved, however, there were higher expectations placed on them. This included obligations to ‘give back’, by using their salaries to build a house in town and to participate in communal activities.

### Citizenship

In the MDP trial, rumours also emerged about blood stealing. The idiom of blood stealing is a narrative that is ever present but is only voiced or realised in unclear or threatening social situations, and medical research conducted by Europeans has historically been experienced as such.^72^ Blood stealing is one of the most common rumours associated with medical research in Africa.^23^^,^^72^^–^^76^ Despite these rumours, women participated in the trial as an act of ‘biological citizenship’, where the biological responsibilities of citizens were embodied in norms of health and health protection.^77^^,^^78^ Biological citizenship is both individualising and collectivising, individualised in that individuals shape their relation with themselves to their biological existence, and collectivised in that individuals organise themselves into specific biomedical classifications, often with specialised medical knowledge of their condition.^79^ This notion of individual and collective biological responsibility was articulated by the women who presented themselves as individuals who took responsibility for their own health by seeking quality healthcare and treatment, as well as the possibility of personal protection from HIV, but also as part of a collective who used their biological existence to test the microbicide gel, for the wider good of society. Citizenship was also expressed through motives to participate. The trial was named Mwanamke Amua kuhusu Maisha yaKO (Woman–decide about your own life) during a consultation with women representatives. In this agreement the intentions of the trial staff converged with women's aspirations for equality and control over HIV prevention for themselves and other Tanzanian women.

The EBOVAC-Salone trial participants also expressed and performed notions of citizenship.^70^ These included expressions of participation as a national sacrifice, to prevent future outbreaks in view of the crisis that had unfolded in the country. Participants’ engagements with the trial also elicited other imaginations of what it ought to mean to be a citizen, as they contrasted the healthcare provided in the trial clinics with that in the government hospital. The trial also entered the political imagination and longstanding negotiations about citizenship through rumours and anxieties that circulated. For example, one rumour emerged that the trial was a pretext to steal blood for foreign use or that participants were injected with Ebola. These were intertwined with anxieties about the epidemic, the role of international actors and government collusion to ‘sell’ its citizens. These were thus ways of expressing deep-seated mistrust and of articulating perceptions of precarity and marginality as Sierra Leonean citizens. At the same time, direct engagement with trial management, including through the advisory modelled on the MDP trial, created new avenues to make demands and assert participants’ rights.

### Uncertainty and hope

In the MDP trial, women articulated uncertainty in everyday life and in the precarious nature of their existence.^80^ They dealt with threats and possibilities every day by drawing on strategies to reduce the chance of negative occurrences and to increase the possibilities of positive ones. These included seeking divine intervention, being brave and seeking knowledge and technology. Their accounts reflected the realities of their economic vulnerability, as well as threats to their physical selves from accidents, violence and disease, especially HIV.^81^^,^^82^ In discussing HIV, the women also constituted themselves as actors of illicit behaviour or as powerless subjects of men's illicit behaviour. They participated in the trial to alleviate their anxiety about HIV infection, however, the uncertainty about the gel's effectiveness accentuated this anxiety. While they understood that the gel's effectiveness to protect against HIV was uncertain, the gel was imbued with hope. Specifically, they ‘hoped’ that it protected them from HIV through its cleansing and lubricating properties.

The EBOVAC-Salone participants also articulated uncertainty in two inter-related ways, to express concerns about the vaccine itself and to relate their experiences of the trial to everyday experience of insecurity. Uncertainty about the vaccine was expressed in the very meaning of a trial, and not knowing whether the vaccine would ‘work’ or whether it would have side effects. For some participants the decision to take part in the trial was not out of conviction that the trial would be safe but rather despite a perception that the risk might be high. This reasoning was not ‘fatalistic’ but rather better understood through notions of destiny and faith that underpin daily efforts to navigate uncertainty. The uncertain outcome of the trial was explained by noting that ‘everything in life is a risk’.

Drawing on experiences of uncertainty and an acceptance of destiny, participants took part in the trial with both apprehension and hope. An acceptance of risk, in other words, also opened up the possibility that one's destiny in the trial could be positive. Hopes were linked to an expectation that the vaccine would work for those who took part in trial, and the possibility that the vaccine would be able to cure other ills and lead to a general improvement in their health. These hopes were not simply misconceptions but a social resource as the trial entered participants’ longer term life trajectories and imaginations.^55^

## Discussion

### Comparative ethnographies

This paper presents a comparative approach across contexts, epidemics and technologies and its value lies in its ability to reveal the salience of history and politics in epidemic contexts, and the role of local understandings of technologies that are implemented to prevent the spread of disease. Exploring narratives from participants in Sierra Leone and Tanzania comparatively, we are able to see how biomedical technologies locate themselves in social imageries in meaningful ways. We identify common themes but trace them as they become enacted in context-specific ways, with different implications for each trial. This comparison, identifying both commonality and specificity, allows us to suggest that these themes may be significant beyond the context in which we have studied them, although their empirical manifestation will differ across communities, diseases and particular technologies and trial designs. This approach underscores the importance of engaging with the social practice of medical research comparatively, setting out an agenda for anthropological contributions to the research of, and engagement with, clinical trials across Africa and beyond.

The emergence of the HIV epidemic in Tanzania revealed the fragility of a healthcare system unable to cope and the reliance on international NGOs for prevention and care. The unequal effects on Tanzanian women's education and employment following structural adjustment exposed their vulnerabilities to HIV infection, especially those working in social venues. Sierra Leone's Ebola emergency was different from Tanzania's HIV crisis because of the different temporality of emergency that made the unprecedented fast tracking of clinical research imaginable. Yet the emergence of Ebola, and later engagements with vaccine trials, also made visible the consequences of an extractive international political economy culminating with the decimation of public healthcare under structural adjustment.

Against this backdrop, our comparative analysis showed the ways in which materiality and exchange are important effects of clinical trials. Clinical trial procedures involve a complex exchange of blood, money, knowledge, drugs and so on between the researchers and those participating in research.^21^ In addition, clinical trials are a productive process, involving time, discomfort, movement and risk.^83^ For the MDP participants, these exchanges were articulated both in material terms, for example, of reimbursement and free healthcare for blood, time and discomfort, as well as immaterial forms of knowledge exchange. EBOVAC-Salone participants similarly focused on blood-giving as a form of sacrifice that elicited expectations of future rewards, both material and immaterial.

In this, social relations were key, both those that predated the trials and made them possible and those that were generated by the arrival of the clinical trial. The MDP trial revealed how social relations were negotiated with women's partners, their communities and the trial staff. The trial, specifically, was part of a wider social and ideological terrain upon which women negotiated gender dynamics, intimacy and sexual meanings.^84^ The trial exposed the women, their partners and local communities to scientific agendas that had moral underpinnings and impacted on the way that sexuality was explored and shaped.^85^^,^^86^ The trial emphasised a medicalised sexuality, separated from the realities of everyday life.^87^ While science frames sexual acts in seemingly amoral biological terms, scientific discourses within the trial had a set of moral assumptions about women's sexual lives, specifically around transactional sex. The trial became a place in which sexuality was negotiated and certain sexual practices such as sex without condoms were discouraged.^87^ The trial also advertently or inadvertently shaped women's sexuality in creative and positive ways, through creating new ideas about equality, pleasure and desire and control over HIV.

The EBOVAC-Salone trial generated different kinds of relations, social imageries and moral economies. As a different technology, the vaccine did not centre on sexuality (although this did arise in relation to contraception requirements), but rather on the meaning of community. Tensions about the boundaries of community and the arrival of ‘strangers’ as trial staff, the political economy of work and of ‘Ebola money’ were countered by the expectations placed on ‘sons of the soil’. These discussions created opportunities to talk about community development and to hold international and state interventions accountable. We explored these tensions and articulations as they emerged through narratives of citizenship and belonging. These narratives also appeared through rumours and anxieties that represent local commentaries to express concern and interpretations of ethics.^23^^,^^72^^–^^74^^,^^88^ The political dimensions of rumours and anxieties around the two trials offered a language to express both disaffection and disillusionment with political life through rumours and anxieties, while at the same time setting the foundations for emancipatory visions of citizenship and deliberation.^70^

### Clinical trial participation and future orientation

Where inequality and poverty dominate, concerns about health and the experience of biomedical technologies ‘seem more about the here and now of medicine than its future possibilities’ [p. 456].^88^ Indeed, our research shows that participants’ experiences materialise through specific political economies and generate social relations and identities in the present. Our research also shows that, just as these biomedical technologies facilitate a particular kind of scientific imagination about managing the future, the gel and the vaccine also entered into trial participants’ hopes, anticipations and future-oriented imaginaries.

Both trials created platforms to discuss the uncertainty of the present. Rather than centre these narratives on misfortunes, trial participants reflected on risk as an openness to possibility.^89^ In contrast to the idea of risk that all future and unexpected events are necessarily negative, uncertainty suggests that future outcomes may also be creative and productive.^90^^,^^91^ For Ådahl, ‘unpredictability or contingency is something that actors engage within their everyday life as an *enabling* aspect of contemporary social experience, leading to creative and varied actions to come to terms with the situation’ [pp. 60–1].^92^ Acting subjunctively with the knowledge that to live is to take risks was accompanied in both sites by a conviction that one's trajectory is determined by powers bigger than ourselves.^89^ Rather than fatalism, this kind of reasoning is best understood through anthropological explorations of ‘destiny’ as a way to make sense of how ‘people imagine and reckon with determining powers’.^93^ An anthropology of destiny allows us to consider how to explore the intersection of a sense of one's ‘capacity, and responsibility to act in their lives’ and ‘worldly and transcendental powers’ [p. 91]. The result is often a malleable fixity, a sense that one makes decisions and navigates terrains that are at least partly preordained. Rather than relinquishing agency to a prewritten fate, then, ideas about destiny underwrite and make it possible to coexist with a feeling of uncertainty.

A key resource for all of the participants in navigating the tension between uncertainty and destiny was hope. Hope was imbued in the technologies themselves, through narratives of protection, well-being and expectations of individual and communal betterment that the trials’ presence was seen to symbolise.^94^^,^^95^ Engagements with biomedicine, in other words, generated new avenues for imagining the future. Although expressed in more open-ended and uncertain terms than the ‘biomedical triumphalism’ of technologies like vaccines, these anticipations were no less hopeful.^5^

### Conclusions

Our comparative ethnographies allow us to highlight the contributions of critical anthropological engagement in clinical trials, taking into account global and local power dynamics while giving space to the voices, imaginaries, hopes and anticipations of trial participants and their communities. These narratives are often hidden by the clinical imagination of the research encounter encapsulated in trial protocols and informed consent forms. A comparative approach that highlights both what is common in the social experience of medical research and how these commonalities are actualised in context-specific ways offers two key contributions. First, it posits a thematic agenda for future research that explores notions of exchange, social relations, political imageries and notions of destiny as domains of a comparative anthropology of medical research across different contexts, technologies, trial designs and diseases. It will be particularly important to develop this comparative agenda to explore how these themes might take on new meaning among participants in therapeutic or intervention trials. Second, it highlights how anthropology can support the design and conduct of clinical trials, expanding our understanding of the social value of clinical research^96^^–^^98^ and showing that trial practices, protocols and technologies do not exist independently of participants’ broader social experiences. Taking participants’ life worlds and deliberations as starting points for research design, rather than as parallel conversations or simply as barriers to ‘acceptability’, can begin to radically reconsider the parameters and standards of medical research.
